# FunMaps: a method for parcellating functional brain networks using resting-state functional MRI data

**DOI:** 10.3389/fnhum.2024.1461590

**Published:** 2024-09-24

**Authors:** Jiayu Shao, Stephen J. Gotts, Taylor L. Li, Alex Martin, Andrew S. Persichetti

**Affiliations:** Section on Cognitive Neuropsychology, Laboratory of Brain and Cognition, National Institute of Mental Health, National Institutes of Health, Bethesda, MD, United States

**Keywords:** resting-state, fMRI, functional connectivity, parcellation, brain networks

## Abstract

Parcellations of resting-state functional magnetic resonance imaging (rs-fMRI) data are widely used to create topographical maps of functional networks in the human brain. While such network maps are highly useful for studying brain organization and function, they usually require large sample sizes to make them, thus creating practical limitations for researchers that would like to carry out parcellations on data collected in their labs. Furthermore, it can be difficult to quantitatively evaluate the results of a parcellation since networks are usually identified using a clustering algorithm, like principal components analysis, on the results of a single group-averaged connectivity map. To address these challenges, we developed the FunMaps method: a parcellation routine that intrinsically incorporates stability and replicability of the parcellation by keeping only network distinctions that agree across halves of the data over multiple random iterations. Here, we demonstrate the efficacy and flexibility of FunMaps, while describing step-by-step instructions for running the program. The FunMaps method is publicly available on GitHub (https://github.com/persichetti-lab/FunMaps). It includes source code for running the parcellation and auxiliary code for preparing data, evaluating the parcellation, and displaying the results.

## Introduction

Functional parcellations of resting state fMRI (rs-fMRI) data provide useful maps of functional networks in human cortex (Glasser et al., 2016; Power et al., 2011; Thomas Yeo et al., 2011). By subdividing the cortex into a topography of functional units, neuroscientists can more easily identify brain areas and networks that are relevant to a particular research question, while also forming new hypotheses based on the network architecture. Furthermore, individual parcels from a functional network map provide a valid means of reducing the number of units in a statistical test (from voxels to parcels) and identifying regions of interest for seed-based connectivity and task-based experiments. Parcellation algorithms can also be used to create group-specific topographies that can be used to compare network characteristics between clinical populations and matched controls (Persichetti et al., 2023). However, creating group-specific functional parcellations is often not feasible with commonly used parcellation methods because they require sample sizes that are much larger than the typical dataset collected in a lab to ensure that the resultant network maps are stable and reliable.

In this paper, we introduce a method, called FunMaps, that we developed to perform flexible and data-driven functional parcellations of the brain to derive network maps with relatively small datasets collected in individual labs. FunMaps incorporates stability measures by searching for networks across random split halves of the data over multiple iterations and then keeping only networks that are present in both halves across several iterations (Persichetti et al., 2021, 2023). The FunMaps parcellation tool comprises several steps that are controlled through a wrapper function. We will walk through the steps of the parcellation, how to enter the desired parameters into the wrapper, and how to evaluate the output at each step. We will show step-by-step results to highlight some of the ways that FunMaps can be applied to rs-fMRI data. In addition, we will describe and evaluate several auxiliary functions that complement the FunMaps method. FunMaps can be downloaded for free on GitHub.^1^ The GitHub page also includes a link to a sample dataset^2^ for users who want to try using the method on data used in this paper.

## Methods

### Demographics

We present data from two separate groups of participants. The main data presented were collected in our lab from 70 typically developing (TD) individuals with no history of psychiatric or neurological disorders [mean (SD) age = 19.7 (3.7) years; 19 female]. We also present data demonstrating how to use the FunMaps method on a restricted region of interest (ROI)—i.e., the anterior temporal lobes (ATL). These data were collected in our lab from 88 individuals with no history of psychiatric or neurologic disorders [mean (SD) age, 21.2 years (7.6 years); 24 females]. Seventy of these participants are from the TD group described above. Subsets of the resting-state data from all of the above individuals have been used in several previous studies (Gotts et al., 2012; Jasmin et al., 2019; Persichetti et al., 2021, 2022, 2023; Power et al., 2019; Ramot et al., 2017). Informed assent and consent were obtained from all participants and/or their parent/guardian when appropriate in accordance with a National Institutes of Health (NIH) Institutional Review Board-approved protocol (10-M-0027, clinical trials number NCT01031407). In addition to the data collected in our lab, we also present data from a sample of 450 participants from the Lifespan Human Connectome Project (HCP) Development project [mean (SD) age = 16.54 (2.94) years, range 12–21 years; 231 female] (Somerville et al., 2018).

### MRI data acquisition and procedure

For all data collected in our lab, scanning was completed on a General Electric Signa HDxt 3.0 T scanner (GE Healthcare) at the NIH Clinical Center NMR Research Facility. For each participant, T2*-weighted blood oxygen level-dependent (BOLD) images covering the whole brain were acquired using an 8-channel receive-only head coil and a gradient echo single-shot echo planar imaging sequence (repetition time = 3,500 ms, echo time = 27 ms, flip angle = 90°, 42 axial contiguous interleaved slices per volume, 3.0 mm slice thickness, 128 × 128 acquisition matrix, single-voxel volume = 1.7 × 1.7 × 3.0 mm, field of view = 22 cm). An acceleration factor of 2 (ASSET) was used to reduce gradient coil heating during the session. In addition to the functional images, a high-resolution T1-weighted anatomical image (magnetization-prepared rapid acquisition with gradient echo—MPRAGE) was obtained (124 axial slices, 1.2 mm^3^ single-voxel volume, 224 × 224 acquisition matrix, field of view = 24 cm). During the resting scans, participants were instructed to relax and keep their eyes fixated on a central cross. Each resting scan lasted 8 min and 10 s for a total of 140 consecutive whole-brain volumes. Independent measures of cardiac and respiratory cycles were recorded during scanning for later artifact removal.

For the HCP Development (HCP-D) data, scanning was completed on 3 T Siemens Prisma scanners (Siemens, Erlangen, Germany). For each participant, T2*-weighted blood oxygen level-dependent (BOLD) images covering the whole brain were acquired using the Siemens 32-channel Prisma head coil and a 2D multiband (MB) gradient-recalled echo (GRE) echo-planar imaging (EPI) sequence (MB8, TR/TE = 800/37 ms, flip angle = 52°) and 2.0 mm isotropic voxels. In addition to the functional images, a high-resolution multi-echoT1-weighted anatomical image (magnetization-prepared rapid acquisition with gradient echo—MPRAGE) was obtained (Harms et al., 2018; Somerville et al., 2018). The MPRAGE scan used a sagittal FOV of 256 × 240 × 166 mm with a matrix size of 320 × 300 × 208 slices. Slice oversampling of 7.7% was used, as was 2-fold in-plane acceleration (GRAPPA) in the phase encode direction and a pixel bandwidth of 744 Hz/Px. Other parameters included: TR/TI = 2,500/1,000, TE = 1.8/3.6/5.4/7.2 ms, flip angle of 8 deg, water excitation employed for fat suppression (to reduce signal from bone marrow and scalp fat), and up to 30 TRs allowed for motion-induced reacquisition. During the resting scans, participants were instructed to stay still, stay awake, and blink normally while looking at the fixation crosshair. Each resting scan lasted 6 min and 30 s for a total of 488 consecutive whole-brain volumes. Independent measures of cardiac and respiratory cycles were recorded during scanning for later artifact removal.

### Preprocessing

All data that were collected in our lab were preprocessed using the AFNI software package (Cox, 1996). First, the initial three TRs from each EPI scan were removed to allow for T1 equilibration. Next, 3dDespike was used to bound outlying time points in each voxel within 4 SDs of the time series mean, and 3dTshift was used to adjust for slice acquisition time within each volume (to *t* = 0). 3dvolreg was then used to align each volume of the resting-state scan series to the first retained volume of the scan. White matter and large ventricle masks were created from the aligned MPRAGE scan using Freesurfer (Fischl et al., 2002). These masks were then resampled to EPI resolution, eroded by 1 voxel to prevent partial volume effects with gray matter voxels, and applied to the volume-registered data to generate white matter and ventricle nuisance regressors before spatial blurring. Scans were then spatially blurred by a 6 mm Gaussian kernel (full-width at half-maximum) and divided by the voxelwise time series mean to yield units of percentage signal change. The data were denoised using the ANATICOR preprocessing approach (Jo et al., 2010). Nuisance regressors for each voxel included the following: six head-position parameter time series (three translation, three rotation), one average eroded ventricle time series, one “localized” eroded white matter time series (averaging the time series of all white matter voxels within a 15-mm radius sphere), eight RETROICOR time series (four cardiac, four respiration) calculated from the cardiac and respiratory measures taken during the scan (Glover et al., 2000), and five respiration volume per time series to minimize end-tidal CO_2_ effects from deep breaths (Birn et al., 2008). All regressors were detrended with a fourth-order polynomial before denoising, and the same detrending was applied during nuisance regression to the voxel time series. Finally, the residual time series were spatially transformed to standard anatomic space (Talairach–Tournoux) at both 2 and 6 mm^3^ isotropic resolutions for computational speed in later analyses.

The HCP-D data were preprocessed using the AFNI software package (Cox, 1996). First, 3dDespike was used to bound outlying time points in each voxel within 4 SDs of the time series mean. Next, unWarpEPI was used to warp posterior-anterior (PA) and anterior-posterior (AP) encoding scans to the midpoint of the two scans (each with 478 TRs), simultaneously accomplishing volume registration. The python script align_epi_anat.py was then used to align the MPRAGE scan to the EPI data. White matter and large ventricle masks were created from the aligned MPRAGE scan using Freesurfer (Fischl et al., 2002). These masks were then resampled to EPI resolution, eroded by 1 voxel to prevent partial volume effects with gray matter voxels, and applied to the volume-registered data to generate white matter and ventricle nuisance regressors before spatial blurring. Scans were then spatially blurred by a 4 mm Gaussian kernel (full-width at half-maximum) and divided by the voxelwise time series mean to yield units of percentage signal change. The data were denoised using the ANATICOR preprocessing approach (Jo et al., 2010). Nuisance regressors for each voxel included the following: six head-position parameter time series (three translation, three rotation), one average eroded ventricle time series, one “localized” eroded white matter time series (averaging the time series of all white matter voxels within a 15 mm radius sphere), and the first 3 PCs of the voxelwise timeseries from the combined white-matter and ventricle masks (modified aCompCor: Behzadi et al., 2007; Stoddard et al., 2016). All regressors were detrended with a fourth-order polynomial before denoising, and the same detrending was applied during nuisance regression to the voxel time series. Following nuisance regression, the PA and AP scans were concatenated, and the residual time series were spatially transformed to standard anatomic space (Talairach–Tournoux) at both 2 and 6 mm^3^ isotropic resolutions for computational speed in later analyses.

## Results

### Brief overview

The FunMaps method is a Matlab based pipeline that also uses AFNI fMRI analysis software (Cox, 1996), and the graph theory-based Infomap algorithm for community detection (Rosvall and Bergstrom, 2008, 2011). Instructions for downloading the FunMaps dependencies can be found on the FunMaps GitHub page. All functions in the main pipeline are controlled through a wrapper function (funmapsWrapper.m) that allows the researcher to update the necessary variables for each step in the pipeline and execute them from one script. Below we will describe each step of the pipeline and which variables coincide with them when applicable (see Table 1 for a schematic of the directory structure and output of FunMaps).

**Table 1:** A schematic of the directory structure that houses the products of the FunMaps method.

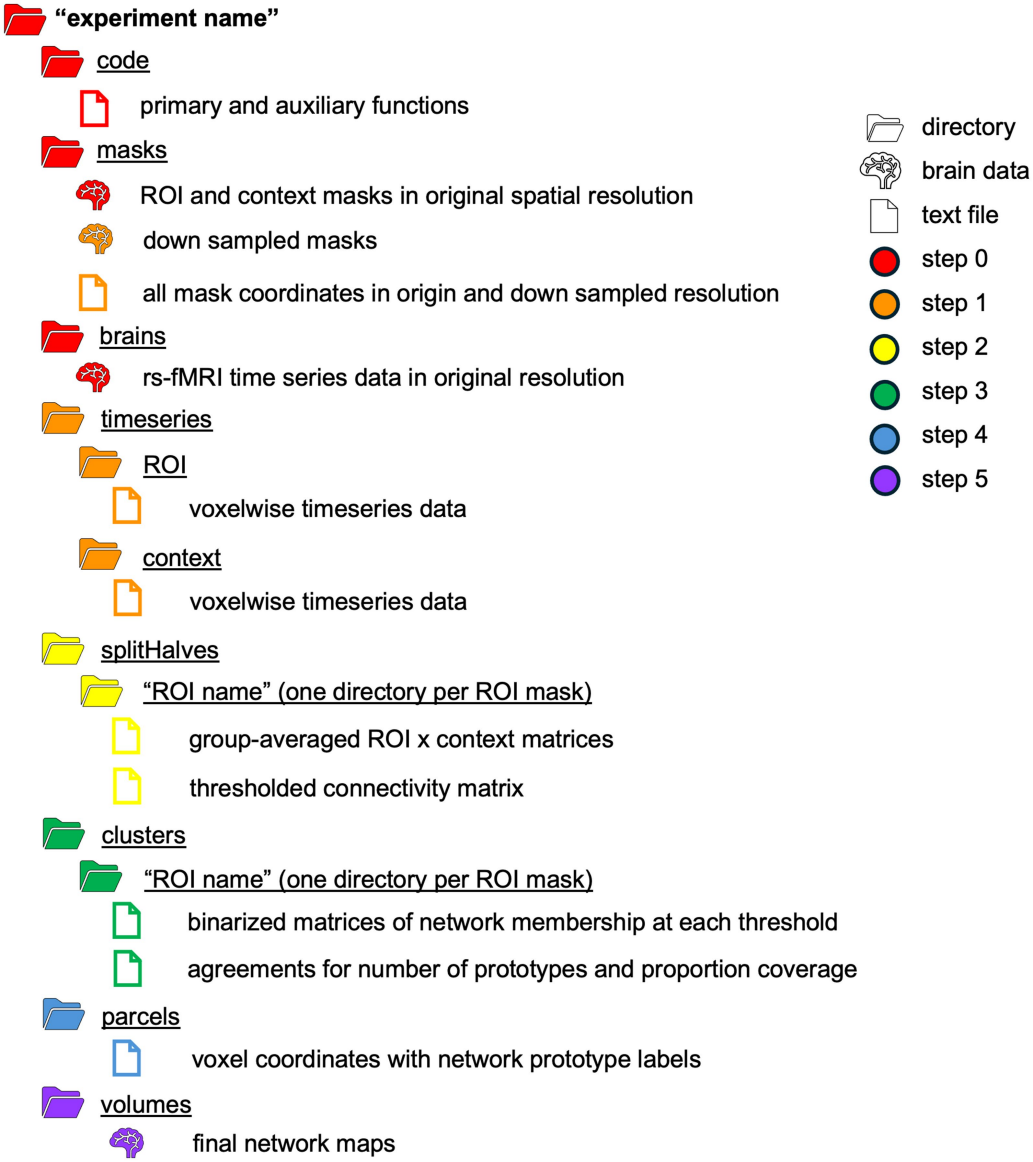

### Step 0: Data and materials needed to run FunMaps

**Figure 1:**
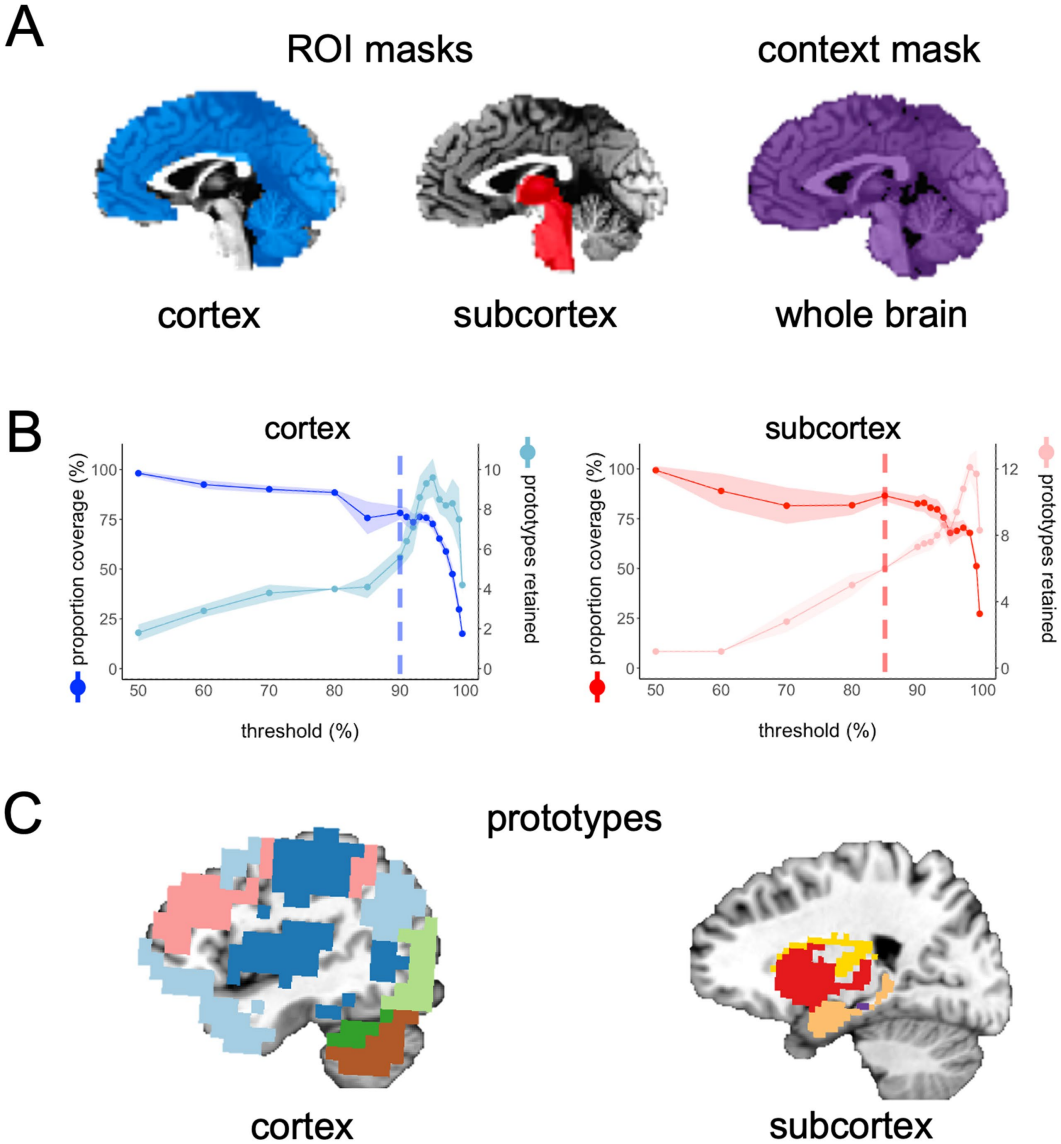
**(A)** In the main example presented in this paper, we chose two ROI masks: a cortical mask that included all cortical and cerebellar voxels (blue) and a subcortical mask that included all voxels in the subcortex (red) and brain stem. Our context mask included all voxels in the whole brain (purple). **(B)** The spilt-half agreement curves were constructed across a wide range of thresholds. The error in the line plots represents ±1 standard deviation across the 10 iterations of the split-half routine. In this example, we chose the 90% threshold in the cortical mask and the 85% threshold for the subcortical mask as the thresholds that maximized proportion of coverage (i.e., number of voxels assigned a network prototype label) and the number of detected network prototypes in each mask (vertical dashed lines). It is critical for the user to evaluate these curves and select thresholds for themselves. **(C)** The cortical network prototypes at the 90% threshold and the subcortical prototypes at the 85% threshold. At this stage of the parcellation, the user can map the prototypes back to the volume at select thresholds to evaluate whether they are acceptable before proceeding to the next steps.

Before running FunMaps, you will need to make an experiment directory with two subdirectories: One called “brains” that contains the cleaned rs-fMRI timeseries data for all participants in the study and another directory called “masks” that contains two types of masks in the native resolution of the timeseries data (Figure 1A). The “brains” directory should contain only one timeseries of equal length from each participant, so the parcellation is not weighted toward an individual or subgroup in the data. The method requires all initial brain data and masks to be in NIfTI format and to be in the users’ preferred standard volumetric space (e.g., our data are in Talairach space—Talairach and Tournoux 1997). The first type of mask is the region of interest (ROI) mask, which consists of all the voxels within the brain region(s) that you want to parcellate. The ROI mask can range in size from a small region of study (e.g., the anterior portion of the temporal lobes—Persichetti et al., 2021) to the whole brain (Persichetti et al., 2023). If your ROI includes both cortical and subcortical voxels, then we recommend separating the ROI mask into cortical and subcortical masks. The pipeline can handle multiple ROI masks at a time, if necessary. The cortical and subcortical ROI’s will be parcellated separately and then combined at a later step in the pipeline. The second type of mask is the context mask, which will often be a whole-brain mask, but you can also decide to exclude voxels that are in your ROI mask—e.g., if you have a small ROI mask and you do not want to use voxel-to-voxel correlations from within the ROI (see Persichetti et al., 2021 for an example). Additionally, we recommend removing voxels with poor temporal signal-to-noise ratio (tSNR) and prominent blood vessel signal from both types of masks. We removed voxels with prominent blood vessel signals by identifying voxels with a relatively high standard deviation in a standard deviation map of the volume registered EPI data (Kalcher et al., 2015).

### Step 1: Extract voxelwise timeseries data from each mask (dumpTS.m)

The *dumpTS* function downsamples the data and masks to a lower spatial resolution, then extracts voxelwise time series data from the rs-fMRI volumes and saves it into text files (Table 1). Downsampling the data before starting the parcellation saves lots of time without sacrificing performance of the parcellation routine, while saving the timeseries data into text files saves space and time, since text files are a small fraction of the file size of a brain volume.

For example, in Persichetti et al. (2023), we started with 2 mm^3^ resolution voxels, then downsampled the whole-brain context mask and the cortical ROI mask to 6 mm^3^ resolution, while the subcortical ROI mask was downsampled to 3 mm^3^ resolution because of its smaller starting volume. Users can choose to omit or modify the degree of down sampling to match the needs of their data, using the variables *roiDownDimArray* and *contextDownDim* in the wrapper. Next, to lower data storage requirements, the ROI and context masks are used to extract voxelwise time series data from the rs-fMRI volumes and save it into 1D vectors. Thus, the output of this step will be new downsampled masks in the *masks* directory (if downsampling is indicated in the wrapper, which we recommended) and a new subdirectory, named *timeseries*, that contains 1D text files of voxelwise rs-fMRI timeseries data from each participant and each mask in the desired spatial resolution (Table 1).

### Step 2: Create random split-half datasets (genSplits.m)

The *genSplits* function randomly splits the participant data into two equal groups, calculates the voxelwise correlation matrices between each ROI mask and the context mask data (done separately for each ROI mask) for each participant, then combines the correlation matrices from all participants in each split-half group to create a group-averaged correlation matrix in each half of the data. This process is repeated over several iterations (we recommend 10 split-half iterations as a good tradeoff between finding stability and minimizing computation time), each time randomly splitting the group of participants into two equal sized groups. The group-averaged ROI × context matrix from each half and each iteration is then made square by calculating the column-wise correlation, yielding an ROI-voxels × ROI-voxels matrix that reflects the similarity of connectivity patterns from ROI voxels to the voxels in the context mask. The final step formats the matrices to be compatible with the InfoMap algorithm that will be used in the next step of the pipeline. Specifically, the real-valued correlation matrices are thresholded into binary (0 or 1) undirected matrices at a range of threshold values representing the top percentages of connections and then converted to the Pajek file format. In the examples used in this paper, we used the following thresholds: 50, 60, 70, 80, 85, 90, 91, 92, 93, 94, 95, 96, 97, 98, 99, and 99.5% (indicated in the wrapper by the variable *testThreshArray* as proportions—e.g., 0.5, 0.6, 0.7, etc.). We used this wide range of thresholds to give the reader a sense of the effect thresholding has on the parcellation routine. However, we recommend that users constrain this range to something closer to steps of 3% between 80–95% to save time, since we have consistently found that ideal thresholds to be 85% for subcortical masks and 90% for cortical masks.

### Step 3: Create network prototypes (genClusters.m)

The *genClusters* function searches for network prototypes in the thresholded matrices of each split-half group using the InfoMap algorithm to form optimal two-level partitions (FunMaps searches for the optimal solution over 100 searches on each split-half iteration). Prototypes found in each half of the data are required to replicate across halves in each iteration. Specifically, in each iteration, a prototype is counted as replicating if the Dice coefficient [( $2|X\cap Y|)∕\left(\left|X|+|Y\right|\right)$ ] is greater than 0.5 and the volume of the intersecting voxels for the prototype is at least 2% of the ROI mask size. Network prototypes that meet these criteria are retained in each iteration. After repeating the above steps for all iterations, an agreement matrix is created, such that each cell reflects the proportion of iterations in which two voxels were part of the same network prototype that agreed across the split halves. Thus, if two voxels were part of a prototype that was present in eight out of 10 iterations, then that cell of the matrix would get a value of 0.8 to indicate that it was present in 80% of the iterations. The matrix is then thresholded such that two voxels are required to be part of the same prototype in at least 50% of the iterations. It is important to note that at this step the matrix has lost the prototype labels and is simply a binarized matrix reflecting generic network prototype membership across voxels. The voxels will be relabeled in the next step of the method (*genParcels*).

The above process is completed for all thresholds indicated in the prior step and agreement curves for each ROI are constructed across thresholds (Figure 1B). The agreement curves can be evaluated to find the threshold with the desired split-half agreement in brain coverage (i.e., the percentage of voxels assigned to a prototype at each threshold) and the total number of prototypes retained. At this point, the program pauses and asks the user to enter on the command line which threshold should be used for each ROI mask. Once the user enters the desired threshold for each ROI mask on the command line, the program resumes the parcellation for those thresholds only. In the example presented here, we chose 90% for the cortical mask and 85% as the threshold for the subcortical mask because we have consistently found these to be ideal thresholds for these types of masks (Figure 1B). In each mask, we consider a threshold “ideal” if both the number of parcels retained and the proportion of coverage are at a “stable” point in the agreement curve (Figure 1B). For example, we chose the 0.85 threshold in the subcortex, because this is the point in the curves where the proportion of coverage is at a local maximum just before it starts a steeper decline (i.e., an unacceptable loss in the number of voxels kept in the mask), while the number of parcels retained is at a relatively flat part of the curve just before a steep increase in the parcels retained that indicates unstable fractionation within the mask. However, it is critical that users evaluate the agreement curves and decide for themselves how they want to proceed, since the optimal threshold will be dependent on features of each dataset, such as sample size and tSNR (the ratio of the average signal intensity to the signal standard deviation across a scan Triantafyllou et al., 2005). In addition to evaluating the agreement curves, the user should run the auxiliary function called *undumpPrototypes.m* that is provided in addition to the core FunMaps method to map the prototypes (in the downsampled space) at a given threshold onto the brain volume (Figure 1C). It is important to note that the number of prototypes that are mapped to the volume may differ slightly from the number indicated on the agreement curve because each prototype must cover at least 2% of the ROI mask. The volumetric prototypes are saved in a text file named with the ROI mask and the threshold value (e.g., cort*ex_prototypeNets_90.1D*) and as a NIfTI formatted brain volume with the same name. The resultant brain map will give the user a good idea about whether the parcellation solution at the selected threshold is reasonable or not.

### Step 4: Assign network labels in the original volume space (genParcels.m)

The *genParcels* function assigns final network labels to each voxel in the original spatial resolution. To save time, this step is completed entirely on vectors in the 1D text file format. The network labels will be mapped onto a brain volume in the next step. First, the program iterates through the timeseries data for each ROI mask in each participant and makes a correlation matrix that reflects the pattern of functional connectivity between each voxel in the ROI mask with all voxels in the context mask. These correlation matrices are then averaged across participants in the downsampled space. These voxelwise patterns of connectivity are then assigned prototype labels, and voxels from the same prototype are averaged together to get an average pattern of brain connectivity for each prototype. The average pattern of brain connectivity for each prototype from all ROI masks is then correlated with the pattern from every voxel across the brain in the original spatial resolution of the data. Thus, a prototype that originated in the subcortical ROI mask can include network voxels in the cortex, and vice versa. In a winner-takes-all approach, each voxel is given the label of the network prototype that explains the most variance in that voxel. However, as a final quality assurance step, the winning network prototype must explain at least 50% of the variance (i.e., *R*^2^ > 0.5) in the functional connectivity pattern of a given voxel for it to get a final network label, otherwise the voxel does not get a label at this step. We do this to avoid giving “noise voxels” (e.g., voxels with prominent blood vessel signal) a network label. At the end of this step, the final network labels are saved in a 1D text file along with the coordinates of all brain voxels in the original spatial resolution of the data. In the next and final step of the program, each voxel will be given a network label while remapping the data into the brain volume.

### Step 5: Create a final volume that includes all networks (genVolume.m)

**Figure 2:**
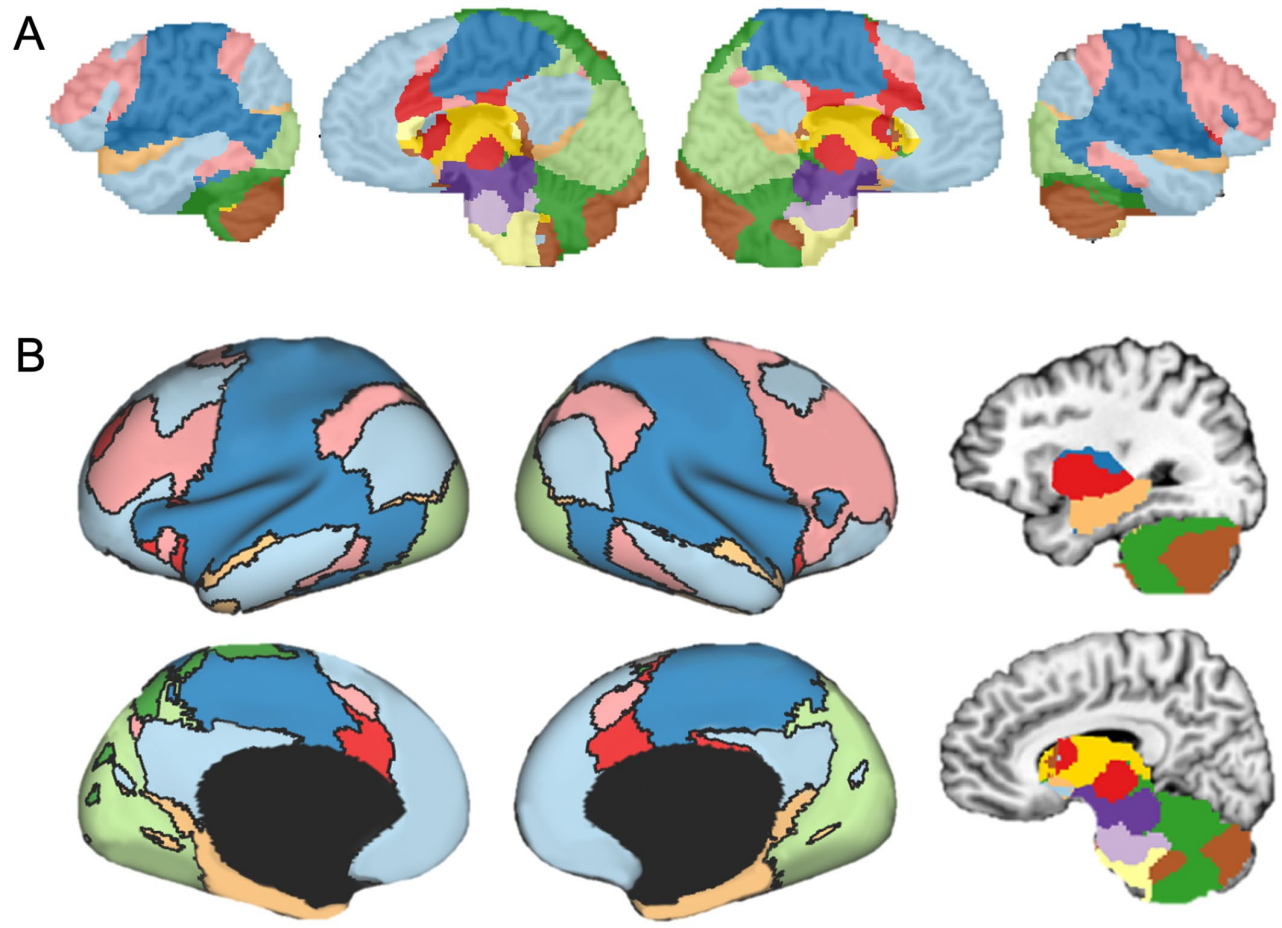
**(A)** The final volumetric network map in the original spatial resolution of the data. **(B)** The auxiliary function called *vol2surf.m* can be used to project the network map onto the surface using the HCP Workbench software.

The *genVolume* function maps the network labels assigned to each voxel in the original spatial resolution of the data onto the brain volume in the NIfTI (.nii) file format. At this step, every voxel that was not assigned a network label in the previous step is given a label using nearest-neighbor interpolation. The map of brain-wide functional networks provided by FunMaps is now complete and the final volumetric rendering of the whole-brain network parcellation can be easily visualized (Figure 2A). The FunMaps GitHub page also includes an auxiliary function called *vol2surf.m* that uses the HCP Connectome Workbench (Marcus et al., 2013) to create a surface rendering of the cortical networks (Figure 2B). Instructions for downloading the Workbench software are on the FunMaps GitHub page.

### Focused parcellations

**Figure 3:**
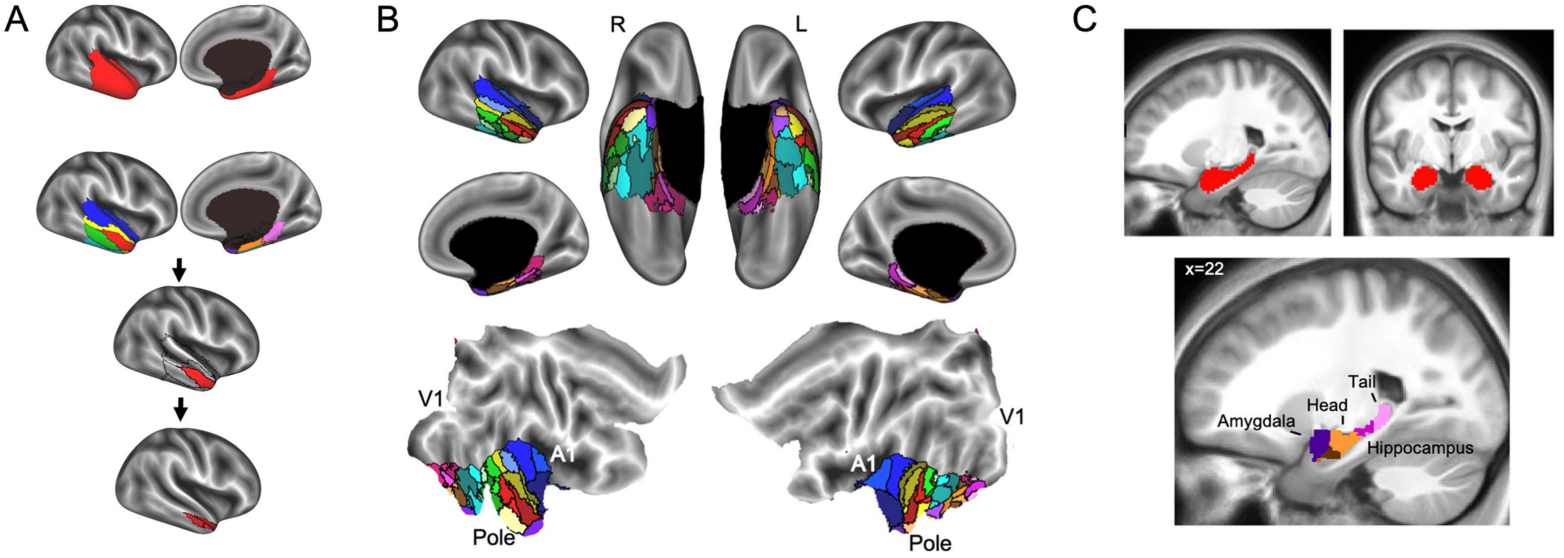
**(A)** The first iteration of the parcellation focused on the cortical (top row) and subcortical masks separately. The cortical mask extended from the anterior tip of the temporal pole back to the Talairach coordinate, *y* = 35. The subcortical mask included the hippocampus and amygdala. Shown here is an example of how to use FunMaps to create an initial parcellation in the ROI mask and then further parcellate each of the parcels in turn to create a more fine-grained network map. After the first pass of the FunMaps routine, the ATL was divided into eight bilateral parcels (second from top row). We then ran FunMaps again on each of the eight parcels, in turn, to further fractionate each parcel (bottom two rows). **(B)** Our parcellation of the ATL resulted in 34 distinct functional parcels based on the patterns of functional connectivity between voxels in the ATL and the rest of the brain. The flat maps in the bottom panel are labeled to orient the reader. V1, primary visual cortex; A1, primary auditory cortex; pole, temporal pole. **(C)** The parcellation within the medial temporal masks (red, top) separated the amygdala (purple) from the hippocampus and further divided the hippocampus into head (orange), body (magenta), and tail (pink). The brown parcel overlaps mostly rhinal cortex and inferolateral hippocampus. This figure is adapted from Persichetti et al. (2021).

In addition to parcellating the whole brain, FunMaps can be used to parcellate select brain regions. In this way, the method can be used to find finer grained parcellations within each network found in the first use of FunMaps. It can also be used to parcellate an *a priori* brain region—e.g., in a recently published paper, we used the FunMaps method to parcellate the anterior portions of the temporal lobes (i.e., the ATL—Persichetti et al., 2021). An example of both uses of FunMaps can be found in the above-mentioned paper, in which we used it to find functional network boundaries in the ATL. Specifically, in that paper, we focused our parcellation on a mask that covered the bilateral ATL and another that included the hippocampus and amygdala (Figure 3). The initial parcellation yielded eight parcels across the cortical and subcortical masks (Figure 3A). We then parcellated each cortical parcel again and ended with a total of 34 distinct functional networks in the ATL (Figure 3B). We provided further analyses to demonstrate that the parcellation identified functionally specific brain networks (see Persichetti et al., 2021 for details). Additionally, results from this paper demonstrated that focusing our parcellation routine specifically on the hippocampus and amygdala identified expected functional boundaries in these structures—separating the amygdala from the hippocampus and further dividing the hippocampus into tail, body, and head (Figure 3C). The divisions within the hippocampus are consistent with an anterior-to-posterior functional gradient within the hippocampus (Fanselow and Dong, 2010; Grady, 2020; Nadel et al., 2013; Poppenk et al., 2013; Ranganath and Ritchey, 2012; Sekeres et al., 2018). Thus, the FunMaps method allows users to control the granularity of the network maps by refocusing the parcellation routine on more restricted brain volumes.

### Sample size

Another major benefit of the FunMaps method is that it requires a very small sample size compared to most parcellation methods. This is excellent news for researchers that want to create maps of functional brain networks that are specific to a dataset that was collected in their lab. This is especially useful for researchers interested in creating functional network maps that are specific to a clinical population. For example, we recently used the FunMaps method and data collected in our lab to create whole-brain functional network maps in a group of 70 individuals with autism spectrum disorder (ASD) and a group of 70 data-quality-matched typically developing (TD) control participants (Persichetti et al., 2023). Each participant in this study completed just one rest run of 8 min and 10 s in the scanner. We were able to identify the most stable parcellation solution in both groups using agreement curves that reflected the proportion of brain coverage and the number of found networks across a series of matrix thresholds. Our ability to find a stable parcellation of the ASD group allowed us to run a series of analyses that suggest patterns of network connectivity between the neocortex and the cerebellum, subcortical structures, and hippocampus are atypical in ASD individuals. Critically, the novel parcellation routine implemented by the FunMaps method allowed us to create a functional network map that was specific to a clinical population using only 70 participants (for comparison, the Thomas Yeo et al. (2011), Power et al. (2011), and Glasser et al. (2016) papers used between 200 and 1,000 participants).

**Figure 4:**
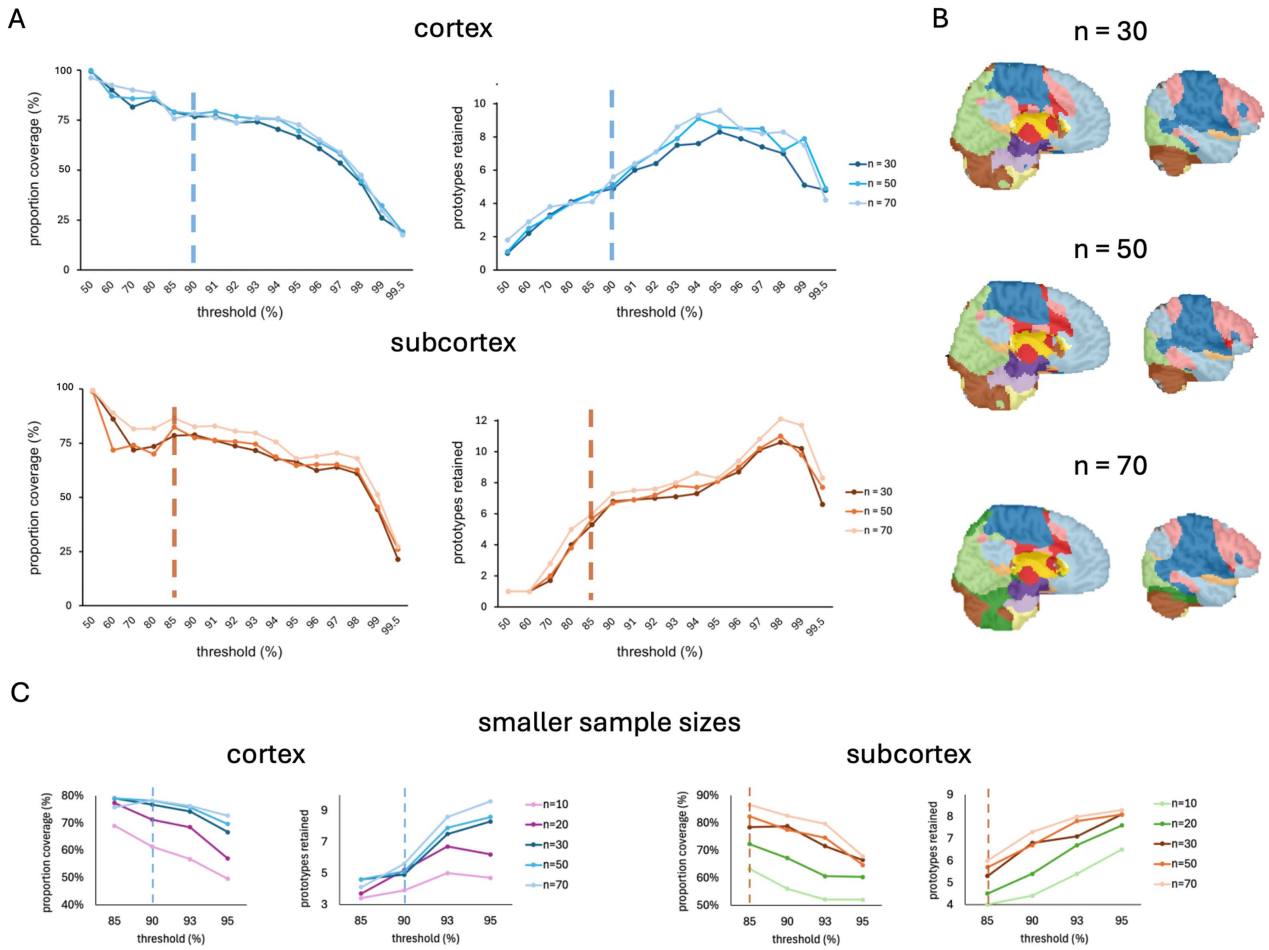
**(A)** The spilt-half agreement curves for sample sizes of 30, 50, and 70 participants. In this example, we chose the 90% threshold in the cortical mask (top) and the 85% threshold for the subcortical mask (bottom) as the thresholds that maximized proportion of coverage (i.e., number of voxels assigned a network prototype label) and the number of detected network prototypes in each mask (vertical dashed lines). **(B)** The final volumetric network maps in the original spatial resolution of the data for each sample size. **(C)** The spilt-half agreement curves for sample sizes of 10 and 20 participants at a restricted range of thresholds (85, 90, 93, 95%) plotted with the data from larger sample sizes. The vertical dotted line indicates the threshold chosen for the parcellation at the larger sample sizes.

In anticipation of the release of the FunMaps method for public use, we asked just how small we can make our sample size and still get a stable parcellation result. We did this by running FunMaps on random subsamples of different sizes from the 70 TD individuals described above. We found stable cortical and subcortical parcellations across subsamples of 30, 50, and 70 participants (Figure 4). These results are very encouraging for our stated goal of offering a parcellation tool that researchers can use on the relatively small sample sizes collected in individual labs. We also ran FunMaps on subsamples of 10 and 20 participants at a restricted range of thresholds (85, 90, 93, 95%) and found that, while these parcellations yielded interpretable results, the parcellation at these sample sizes yielded less network prototypes and an appreciable reduction in coverage compared to results from larger sample sizes in both cortex and subcortex (Figure 4C). Therefore, we did not proceed further with the parcellation at these subsamples. However, this does not mean that the method is not usable with sample sizes smaller than 30 participants. Data quality and length of the rs-fMRI run time play critical roles in identifying stable functional networks in the brain (Gonzalez-Castillo et al., 2014). Thus, collecting more timepoints of rs-fMRI, acquiring data in higher field strengths (e.g., 7 Tesla), and using more sophisticated acquisition sequences (e.g., multi-echo acquisitions—Cohen et al., 2021) are good ways to increase the likelihood that FunMaps will find optimal functional networks in your brain data (Laumann et al., 2017; Lynch et al., 2020). While we have demonstrated that FunMaps can be used to obtain functional network maps in small groups of individuals, it is worth noting that the method can also be used to find individual-specific network maps, so long as enough data are collected per study participant (e.g., Gordon et al., 2017).

**Figure 5:**
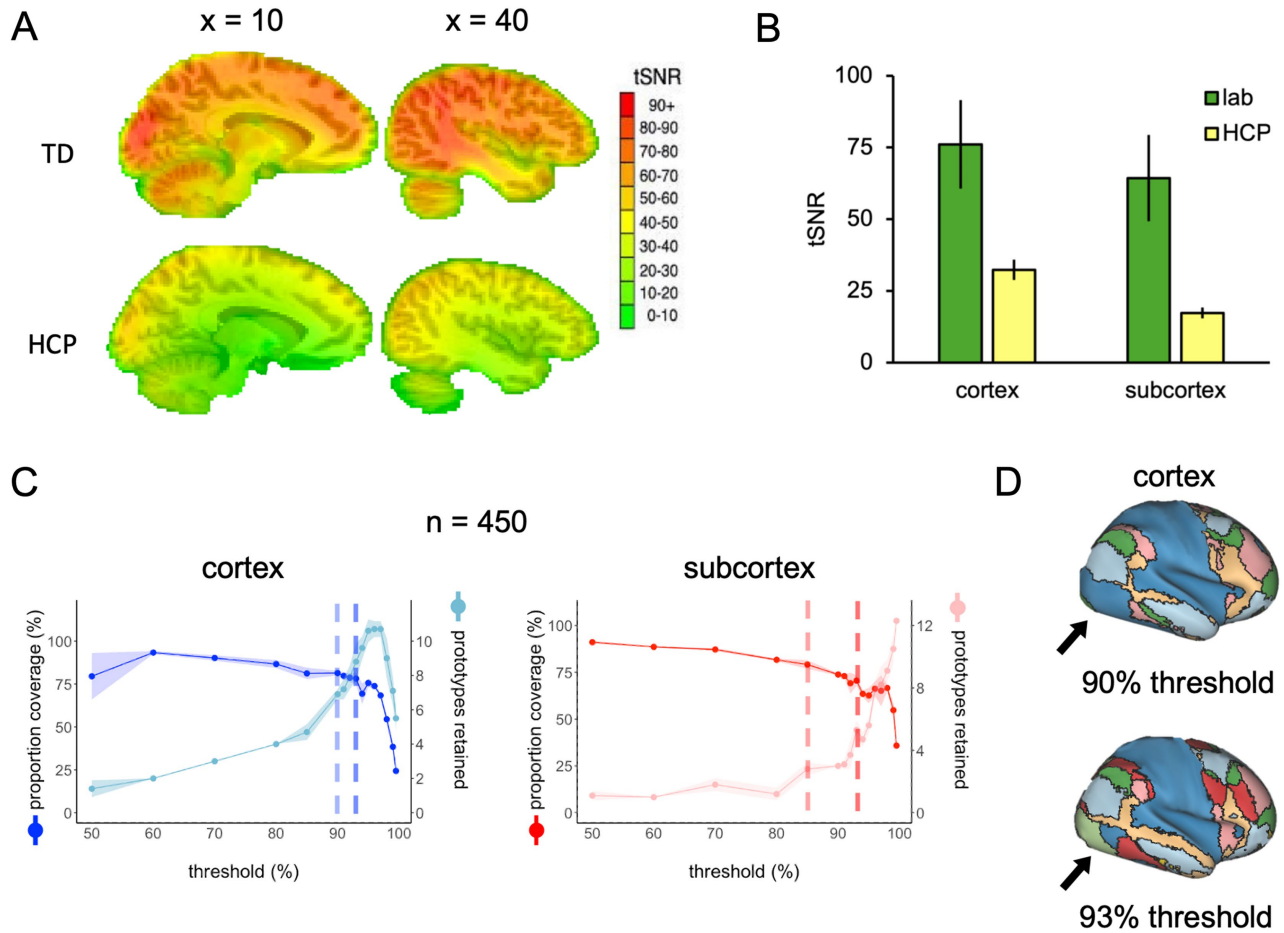
**(A)** Whole-brain tSNR maps averaged across 70 participants from our lab (top) and 450 HCP participants (bottom). **(B)** The tSNR values averaged across the cortical and subcortical masks separately in the 70 participants from our lab and the 70 highest tSNR values from the HCP data. **(C)** The spilt-half agreement curves across a wide range of thresholds. The error in the line plots represents ±1 standard deviation across the 10 iterations of the split-half routine. The agreement curves in the cortex are a good example of why it is important to check the protoypes in the brain volume along with looking at the curves. At the 90% threshold (lighter vertical dashed line), the cortical prototypes are reasonable and similar to the results from our lab example, except that visual regions in the occipital lobes do not separate from the sensorimotor areas in the parietal lobe. At the 93% threshold (darker vertical dashed line), the prototypes look very similar, except now the visual and sensorimotor areas in the occipital and parietal lobes, respectively, are now separate prototypes. Meanwhile, the algorithm did not detect any subcortical network prototypes that met our inclusion criteria until the 85% threshold, then the number of prototypes increased rapidly at subsequent thresholds. **(D)** When the functional networks are mapped to the cortical surface at the 90 and 93% thresholds, it is clear to see that the visual regions in the occipital lobe do not separate from the sensorimotor areas in the parietal lobe at the 90% threshold, but do separate at the 93% threshold.

The results described thus far are a product of applying FunMaps to rs-fMRI data with good tSNR values across the whole brain. To demonstrate how the parcellation routine performs on rs-fMRI data with much lower tSNR, we ran FunMaps on a group of 450 randomly selected participants from the HCP database. First, we measured the tSNR across the HCP participants and compared it to the 70 participants that were collected in our lab (Figure 5A). The tSNR was significantly lower in the HCP data compared to our data (Figure 5B). Even when using an unpaired *t*-test to compare the 70 highest tSNR values from the HCP data with our data in cortex and subcortex separately, the difference is pronounced (cortex_(138)_, *t* = 47.85, *p* < 10^−4^, subcortex_(138)_, *t* = 68.84, *p* < 10^−4^). While this significant reduction in tSNR influenced our ability to find stable functional networks in the HCP data, especially in the subcortex, we still achieved reasonable results.

The agreement curves for the cortical and subcortical masks provide good examples of why it is crucial that users look at the prototypes mapped onto the brain volume (Figure 5C). In the cortical mask, the agreement curves are similar to the agreement curves we get in our lab data (displayed in Figure 1B). However, when we mapped the prototypes to the brain volume, we found that at the 90% threshold (lighter vertical dashed line in Figure 5C), the cortical prototypes are reasonable and similar to the results from our lab example, except that visual regions in the occipital lobe do not separate from the sensorimotor areas in the parietal lobe. At the 93% threshold (darker vertical dashed line in Figure 5C), the prototypes look very similar, but now the visual and sensorimotor areas in the occipital and parietal lobes, respectively, are separate prototypes (Figure 5D). Thus, the 93% threshold is likely the better choice for these data.

**Figure 6:**
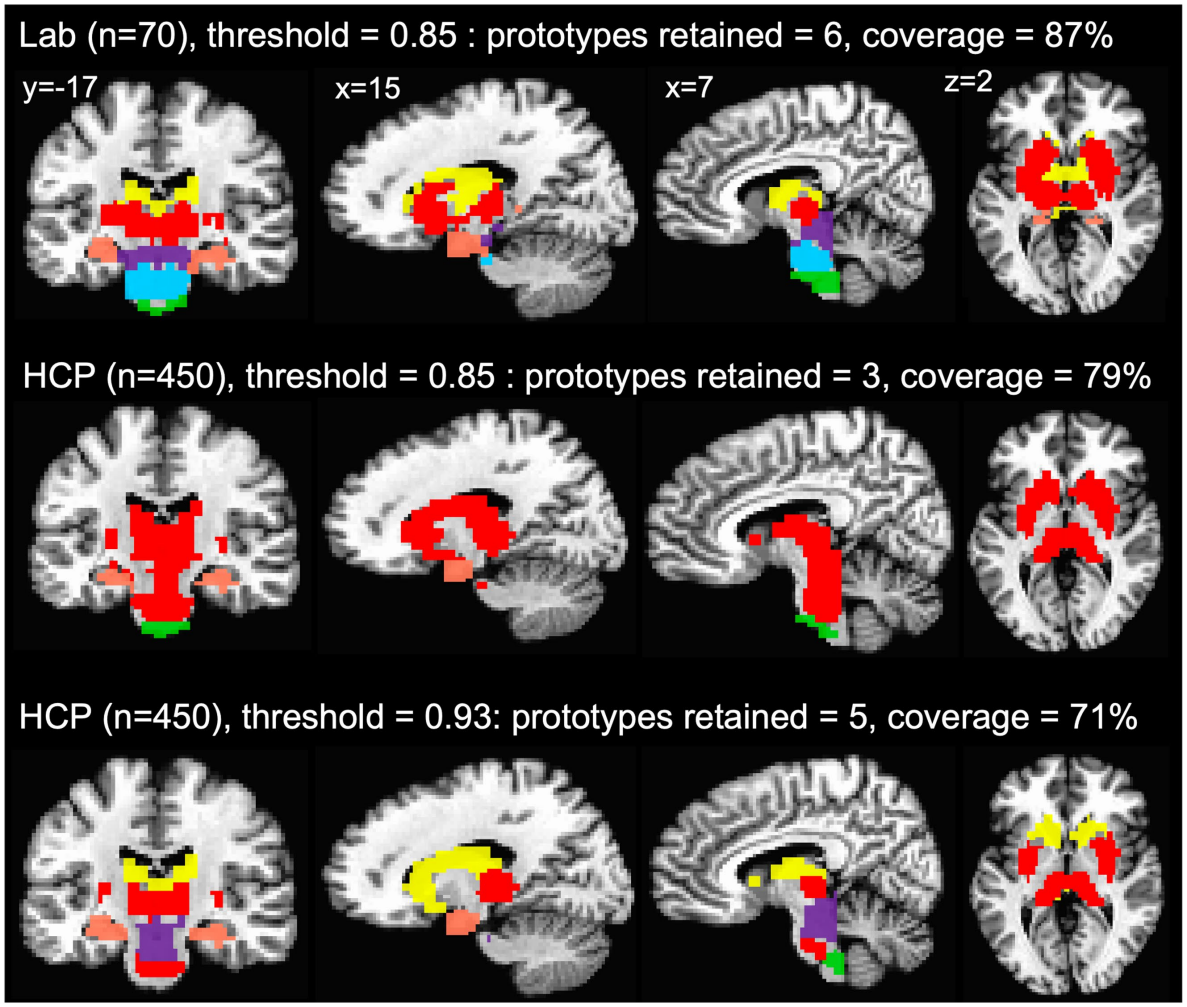
Network prototypes identified at the 85% threshold in the lab data (top row) and HCP data (middle row), and at the 93% threshold in the HCP data (bottom row). In the HCP data, the proportion of coverage at the 85% threshold is a bit lower than the lab data, and there are only three prototypes that separate the hippocampus and medulla from everything else (middle row). In the HCP data, the retained prototypes at the 93% threshold (bottom row) are similar to those found in the lab data (top row) with the exception that the HCP data does not demarcate the pons. However, at the 93% threshold almost a third of the voxels in the subcortical mask are not assigned a network label.

The agreement curves from the subcortical mask in the HCP data are also similar to the agreement curves we get in our lab data, but with a few important differences that become even clearer when we evaluate the network prototypes in the brain volume. In the HCP data, the proportion of coverage at the 85% threshold is a bit lower than the lab data (79% vs. 87% in the lab data—Figure 5C) and it is clear that there are fewer voxels filled in the HCP brain compared to the lab data (Figure 6, top and middle rows). The bigger problem with the parcellation solution at the 85% threshold is that there are only three prototypes that separate the hippocampus and medulla from everything else—probably too coarse to be useful (Figure 6, middle row). By contrast, in the lab data, there are six prototypes that map subcortical structures reasonably well (e.g., it demarcates the hippocampus and separates the brainstem into midbrain, pons, and medulla—Figure 6, top row). We can also see in the agreement curve for the lab data that the number of prototypes starts to rise from a baseline small number (~2) by the 70% threshold (Figure 1B). By contrast, in the HCP agreement curve the number of prototypes does not start to rise from the baseline small number (~2) until the 85% threshold (light dashed vertical line) and remains relatively low until around the 93% threshold (dark dashed vertical line—Figure 5C). It is not unreasonable in this situation to evaluate the parcellation at the 93% threshold, since there are more prototypes identified at that point. In the bottom row of Figure 6, we can see that the retained prototypes are similar to those found in the lab data with the exception that the HCP data does not demarcate the pons. However, it is also clear that the proportion of coverage (71%) is now even worse, with almost a third of the voxels in the subcortical mask not being given a label. The 29% of unlabelled voxels in the HCP subcortical mask is over double the unlabelled voxels in the Lab data (13%). This lack of coverage is concerning because it is an indication of instability in the connectivity patterns of these voxels. Furthermore, it can lead to problems at the next step of combining the cortical and subcortical prototypes and assigning a final network label to all voxels in the brain, since that step is really meant to fill small patches of unlabelled voxels rather than large numbers of voxels. That said, the parcellation at the 93% threshold is still useful since it identifies subcortical structures reasonably well. A researcher could stop here and use these network prototypes as regions of interest or seeds, if it suits them. We do recommend caution when proceeding to the next step of the parcellation, if there is a large number of unlabelled voxels.

It is important to note that our intention for applying FunMaps to the HCP data was to demonstrate its flexibility by showing that it produces reasonably similar network parcellations across diverse datasets. We used a subset of 450 HCP participants, but there are over 1,200 participants in the HCP database and we are confident that the parcellation in the HCP data would improve by including more participants. We also want to make clear that by pointing out that the HCP data have significantly lower tSNR than our lab data, we do not wish to imply that the HCP data are bad (or our data are unusually good). Rather, we are firm believers that the HCP, and other large data collection initiatives like it, are highly valuable to the field of cognitive neuroscience.

## Conclusion

We introduced the FunMaps method as a flexible and easy-to-use way of creating topographical maps of functional networks in the human brain using relatively small rs-fMRI datasets that are collected in individual labs. In this paper, we have demonstrated how FunMaps can be used to create stable and replicable multi-level network maps across the whole brain or in focused regions of interest. We developed the FunMaps method to be flexible and easy to use. It is publicly available on GitHub (see text footnote 1) and includes source code for running the parcellation; auxiliary code for preparing data, evaluating the parcellation, and displaying the results; and further documentation on how to use the method.

## Data Availability

The datasets presented in this study can be found in online repositories. The names of the repository/repositories and accession number(s) can be found at: https://osf.io/we8k3.

## Footnotes

^1^https://github.com/persichetti-lab/FunMaps

^2^https://osf.io/we8k3
